# Reproductive performance in pigs reared under organic conditions compared with conventionally reared pigs

**DOI:** 10.1186/1751-0147-55-33

**Published:** 2013-04-17

**Authors:** Ylva Lindgren, Nils Lundeheim, Sofia Boqvist, Ulf Magnusson

**Affiliations:** 1 Division of Reproduction, Department of Clinical Sciences, University of Agricultural Sciences, P.O. Box 7054, Uppsala SE-750 07, Sweden; 2 Department of Animal Breeding and Genetics, University of Agricultural Sciences, P.O. Box 7023, Uppsala, SE-750 07, Sweden; 3 Department of Biomedicine and Public health, University of Agricultural Sciences, P.O. Box 7028, Uppsala, SE-750 07, Sweden

**Keywords:** Pigs, Reproduction, Organic farming

## Abstract

**Background:**

To achieve a competitive reproductive performance in organic pig farming is a major challenge for this farming practise. Practices and research data regarding conventional pig production are not always applicable to organic production, why field studies are needed to identify differences in performance between organic and conventional pig farms in order to identify areas for improvement.

**Results:**

Performance data for one year was collected from 5 organic herds that had more than 30 sows in production and used a computerized recording system, and data from five nearby conventional farms with more than 30 sows and the same recording system were used as a comparison. In total data from 4697 farrowings were analyzed. In the organic pig herds, there were a higher total number of piglets born per litter (p=0.001), a higher number of piglets stillborn per litter (p<0.001), but a tendency (p<0.06) to lower number of weaned pigs per litter and longer nursing period (p<0.001) and farrowing interval (p<0.001).

**Conclusions:**

The reproductive performance was lower in the organic herds and the variation in reproductive performance among the organic herds was larger than among the conventional ones, suggesting options for improvement in the organic herds.

## Background

There are EU-regulations [1] as well as national requirements that must be fulfilled in order to receive national organic certification, which is the case in for example Sweden [2]. In Sweden the most prominent features of certified organic production are that: 1) the pigs have to have access to outdoor areas (pasture during summertime, paddocks during wintertime) 2) that several sows and their piglets must be group-housed during the nursing period from 2 weeks post-farrowing and onwards, 3) the nursing period must be at least 40 days. Given these requirements, it is major a challenge to obtain a reproductive performance similar to conventional pig farming.

The underlying factors for this challenge is that group-housing of sows during lactation is known to lead to an increased proportion of sows ovulating during this period, largely as a consequence of a decreased nursing intensity, especially in older sows [3]. Also, as the weaning age of the piglets is higher according to the Swedish organic regulations compared with the minimum requirement of 28 days in conventional Swedish pig farming, this contributes to a more gradual weaning and decrease in nursing intensity [4,5]. The ovulations during lactation lead to a time-wise more scattered distribution of sows showing oestrus and thus a prolonged subsequent breeding period [3,6], with reduced possibilities to run a strict batch-wise production. Obviously, practices and research data regarding conventional pig production are not always applicable to organic production. Solid field studies on reproductive performance of sows in organic production are sparse. Therefore, in the present study we compare field data on reproductive performance in organic pig herds with those from conventional pig herds, and discuss areas for improvement.

## Materials and methods

Five organic piglet-producing farms fulfilled the criteria for being included in the study and five conventional commercial farms were chosen as comparison. The criteria to be fulfilled by the farms, in order for them to be included in the study were: the farms should have more than 30 sows in production, they should use the computerized performing monitoring system PigWin Sugg (Svenska Pig) and, obviously, they should have owners who were willing to share their herd data. The organic farms adhered to the Swedish requirements for organic certified production [2].

The certified organic farms were the five among the 21 piglet producing organic farms serviced by the Swedish Animal Health Service that fulfilled the criteria above. Five conventional piglet-producing farms were chosen as a comparison to the five organic farms. To each of the organic farms, the owner of the conventional farm geographically located the closest and using PigWin Sugg was asked to participate. In the conventional farms, the sows and their piglets were housed in individual pens throughout the nursing period. In both farm types cross-fostering was applied in order to even out the sizes of the litters.

The data extracted from the herds’ computerized performance monitoring system (PigWin Sugg, Svenska Pig), consisted of information on farrowings in the period April 2007 to March 2008. Also, data of the sows’ next farrowing, after this time period, was captured from the data in order to calculate farrowing interval. Data was transferred to the SAS software (ver. SAS 9.2; SAS Institute Inc., Cary, NC, USA) for editing and analysis. The number of farrowings per organic farm ranged from 72 to 392 and the corresponding number per conventional farm ranged from 217 to 1288. The total number of farrowings, both categories, during the 12 months was 4697, 830 in organic herds and 3867 in conventional herds, giving a total number of 60786 piglets born. In both types of herds, the sow was crossbred between Landrace and Yorkshire, and the piglets were sired by Hampshire AI boars. Notably, the genetic background for pigs in organic production in Sweden is the same as in conventional pig production [7].

Data was analysed using analysis of variance (PROC MIXED) in the SAS software (ver. SAS 9.2; SAS Institute Inc., Cary, NC, USA). The analysed traits were for each litter: total born, live born and stillborn piglets, and number piglets weaned, weaning age and following farrowing interval. Farrowing intervals longer than 300 days were in the analyses regarded as missing observations. The statistical model included the fixed effects of herd type (organic or conventional), herd nested within herd type, two-month periods of farrowing and parity number (1, 2, 3, 4+). The statistical model also included the random effect of sow, nested within herd and herd type. Least squares means were calculated, and pair-wise comparisons were made using Student’s t-test.

## Results and discussion

In the organic herds the total number of piglets born per litter, (p <0.001), and the number of piglets stillborn per litter, (p<0.001) was higher than in the conventional herds, +0.8 and +0.4, respectively (Table 1). Possibly, one contributing factor to this would be the longer nursing period in the organic system, which implies a longer recovery period for the sow before next mating which has been shown to be beneficial for the reproductive performance [8].

**Table 1:** Comparison of reproductive performance (LS means and range between farms) between 5 organic and 5 conventional Swedish piglet producing herds (n=4697 farrowings; whereof 830 in the organic herds and 3867 in the conventional herds)

**Trait**	**Herd type**		***p*-value**
	**Organic LS means (Range)**	**Conventional LS means (Range)**	
**Total number of piglets born per litter**	13.6 (12.7-13.8)	12.8 (12.2-13.5)	0.0006
**Number of piglets born alive per litter**	12.4 (10.8-12.9)	12.0 (11.7-12.6)	0.6
**Number of piglets stillborn per litter**	1.2 (0.9-2.2)	0.8 (0.5-0.9)	<0.0001
**Number of piglets weaned per litter**	9.8 (9.0-10.8)	10.0 (9.7-10.4)	0.06
**Length of nursing period (=age at weaning) in days**	50 (40–58)	34 (33–39)	<0.0001
**Farrowing interval in days**	178 (169–191)	158 (156–165)	<0.0001

**Figure 1:**
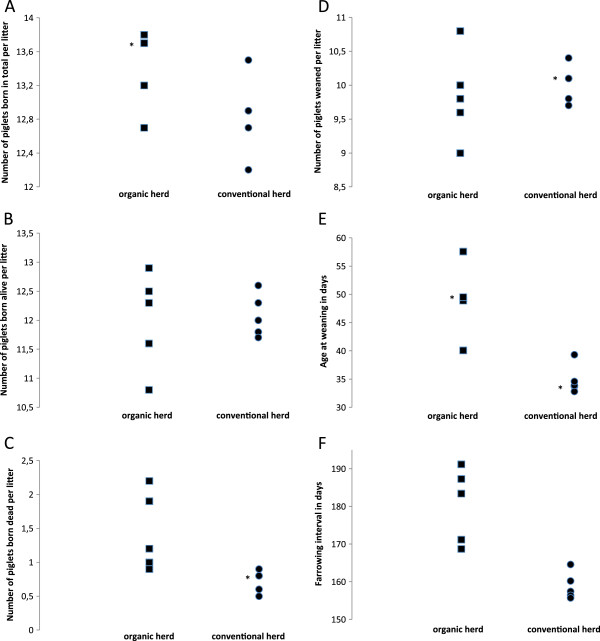
**LSmean for total number of piglets born per litter (panel 1A), number of piglets born alive per litter (panel 1B), number o piglets born dead per litter (panel 1C), number of piglets weaned per litter (panel 1D), age at weaning in days (panel 1E), farrowing interval in days (panel 1 F) in five organic (squares) and five conventional (circles) herds.** Asterix indicate that there are two herds with the same value.

In contrast, the number of piglets born alive per litter did not significantly differ between herd types but for this trait there was a large variation among the organic herds (Figure 1B). This might reflect larger variations among the organic hers in housing and management. The number of piglets weaned per litter tended (p=0.06) to be higher in the conventional herds, indicating a higher pre-weaning mortality in the organic herds. Notably, previous studies have shown higher piglet mortality in organic herds [9] and non-organic group-housing system [10] compared to conventional herds with sows kept individually during lactation. One possible reason for this is the opportunity for the loose-housed sow to escape from her piglets [5], which may lead to inadequate nursing and weakening of the piglets [11]. Also, crushing of the piglets by the sow during the first days of life has been reported to be a more common cause of death in organic than in conventional herds [9].

In the organic herds the nursing period was 16 days longer (p<0.001) than in the conventional herds. Interestingly, the mean age at weaning was 50 days in the organic herds, which is 10 days longer than the minimum requirement [2] and 34 days in the conventional herds, which is 6 days longer than the minimum requirement in the Swedish animal welfare regulations [12]. These findings suggest a possibility for both types of farms to lower the age at weaning and thus increase the number of litters per sow and year (Figure 1E).

In the organic herds the farrowing interval were 20 days longer (p<0.001) than in the conventional herds. Most of this difference (16 days) can be explained by the longer nursing period in the organic herds. The difference of the additional four days might be explained by later and more scattered estrous among the organic sows. Further, the variation in lengths of farrowing intervals was greater among the organic herds than among the conventional herds and there was one organic herd with a farrowing interval similar to that in conventional herds (Figure 1F).

Finally, within each herd type there was a significant (p<0.01) effect of herd (Figure 1), and parity number for all six analyzed reproductive traits.

## Conclusion

The data from this study should be regarded as indicative as just five organic herds fulfilled the criteria enabling a systematic comparison with conventional herds. Even so, the results illustrate that the reproductive performance differ considerably between organic and conventional pig production in Sweden. The variation in reproductive performance among the organic herds is larger than among the conventional ones, suggesting options for improvement in the former type of herds.

## Competing interests

The authors declare that they have no competing interests.

## Authors’ contributions

YL collected and organized the data and drafted the manuscript. NL participated in the planning of the study, performed the statistical analysis and commented on the manuscript. SB participated in the planning of the study, assisted in the organization of the data and statistical analysis and commented on the manuscript. UM organized the study, commented on the statistical analyses and finalized the manuscript. All authors read and approved the final version of the manuscript.
